# Modelling imperfect adherence to HIV induction therapy

**DOI:** 10.1186/1471-2334-10-6

**Published:** 2010-01-12

**Authors:** Rachelle E Miron, Robert J Smith?

**Affiliations:** 1 Department of Mathematics, The University of Ottawa, 585 King Edward Ave, Ottawa ON K1N 6N5, Canada; 2 Department of Mathematics and Faculty of Medicine, The University of Ottawa, 585 King Edward Ave, Ottawa ON K1N 6N5, Canada

## Abstract

**Background:**

Induction-maintenance therapy is a treatment regime where patients are prescribed an intense course of treatment for a short period of time (the induction phase), followed by a simplified long-term regimen (maintenance). Since induction therapy has a significantly higher chance of pill fatigue than maintenance therapy, patients might take drug holidays during this period. Without guidance, patients who choose to stop therapy will each be making individual decisions, with no scientific basis.

**Methods:**

We use mathematical modelling to investigate the effect of imperfect adherence during the inductive phase. We address the following research questions: 1. Can we theoretically determine the maximal length of a possible drug holiday and the minimal number of doses that must subsequently be taken while still avoiding resistance? 2. How many drug holidays can be taken during the induction phase?

**Results:**

For a 180 day therapeutic program, a patient can take several drug holidays, but then has to follow each drug holiday with a strict, but fairly straightforward, drug-taking regimen. Since the results are dependent upon the drug regimen, we calculated the length and number of drug holidays for all fifteen protease-sparing triple-drug cocktails that have been approved by the US Food and Drug Administration.

**Conclusions:**

Induction therapy with partial adherence is tolerable, but the outcome depends on the drug cocktail. Our theoretical predictions are in line with recent results from pilot studies of short-cycle treatment interruption strategies and may be useful in guiding the design of future clinical trials.

## Background

Currently, 33 million people worldwide are infected with HIV/AIDS, of whom 2.7 million were infected in 2007 [1]. HIV is a disease that is accompanied by a profound depletion in the number of CD4^+ ^T cells and can be transmitted by blood or other body fluids [2]. Most patients with HIV/AIDS are prescribed a triple-drug cocktail with either three nucleoside-analogue reverse transcriptase inhibitors (RTIs), or two RTIs and one protease inhibitor (PI) [3]. However, PI-sparing cocktails have been shown to have equivalent potency to PI-containing cocktails [4] and may reduce the risk of metabolic and potential cardiovascular consequences of PI-containing therapy, while providing similar or improved virologic control and durability of effect [5].

The importance of adherence to HIV drug regimens presents challenges that arise from the biology of HIV, the magnitude of the required therapeutic effort and the changing demography of HIV infection [6]. In order to determine regimens for partial adherence, a number of mathematical models have attempted to quantify how drug concentration levels in the body of an HIV patient affect viral replication [7-13].

Adherence to drug therapy is necessary in order to control HIV, but sometimes-overwhelming side effects, as well as the inconvenience of following a strict regimen, deter patients from taking their drugs [14]. Imperfect or partial adherence can facilitate the emergence of drug-resistant mutations [15].

Induction therapy is a HIV/AIDS treatment regime that hopes to benefit patients by decreasing drug resistance and reducing the overall number of drugs that must be taken. In order to minimise drug resistance, induction-maintenance (IM) therapy strategies begin with a period of intensified antiretroviral therapy (induction phase), followed by a simplified, long-term regimen (maintenance phase) [16-19].

Previous work with induction therapy failed due to uncalculated latently infected cells and imperfect adherence [17,19]. Recently, however, Curlin *et al*. [20] have shown that a longer induction phase decreases the probability that viruses resistant to maintenance therapy will emerge. Their studies have shown that the probability of success (maintaining a suppressed, circulating, free-virus population for a period of at least 3 years after the end of induction therapy) varies with the length and time of the induction phase [20]. Using a stochastic model, it was shown that induction therapy would have to last at least 180 days for cocktails containing two RTI-like drugs and a PI-like drug [20,21].

Imperfect adherence has led to failure in suppressing viral replication and often mutations develop before or during induction therapy [6]. Since induction therapy has a significantly higher chance of pill fatigue than maintenance therapy, it is likely the patients will take some holidays during this period. Scientific literature cautions patients against taking any holidays while on therapy [22], but many patients are underadherent or nonadherent [23,24]. Without guidance, patients who choose to stop therapy will each be making individual decisions, with no scientific basis. Recently, the question of short-term holidays (such as weekends) have been examined. Patients were highly adherent to five days on/two days off (FOTO) therapy. When asked about their preference for this type of therapy versus continuous HAART (Highly Active Antiretroviral Therapy), on a 10-point scale, the mean response was 9.7 [25].

Here, we examine the effects of imperfect adherence during the induction phase using a mathematical model of impulsive differential equations. We use the model to address the following research questions: 1. Can we determine the maximal length of a drug holiday and the number of subsequent doses that must be taken to avoid resistance? 2. How many drug holidays can be taken during the induction phase?

## Methods

### Modelling drug therapy

When modelling drug therapy and trying to approximate the number of doses a patient can miss without gaining drug resistance, it is important to have a reliable threshold that will guarantee that viral replication will not exceed a safe limit and so that the mutant strain will not appear. The inhibition of viral replication, *s*, can be described by

where *R*(*t*) is the drug and *IC*_50 _is the concentration of drug which inhibits viral replication by 50% [7].

**Figure 1:**
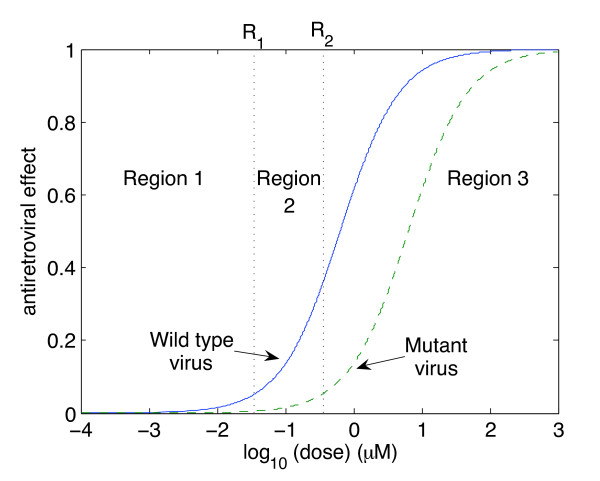
**Dose-effect curves**. Example of dose-effect curves for the wild-type (solid blue curve) and 10-fold resistance (dashed green curve) virus strains. When drug concentration levels are in Region 1, the amount of drug is insufficient to either control wild-type or mutant strains. When drug concentration levels are in Region 2, the amount of drug is sufficient to block the wild-type virus but resistant virus may emerge. When drug concentration levels are in Region 3, both virus strains are controlled. This example is for the reverse transcriptase inhibitor Stavudine (d4T).

Thus, when *s *≈ 0, the drug has no effect, while if *s *≈ 1, the drug completely inhibits viral replication. See Figure 1.

Thus, the antiretroviral drug effect can be split into three regions: in Region 1, drug levels are insufficient to control either the wild-type or the mutant strain. In Region 2, drug levels are sufficient to control the wild-type strain but not a 10-fold mutant strain of the virus (ie a mutant strain that requires ten times the amount of drug to be controlled). In Region 3, drug levels are sufficient to control replication of both virus strains. These findings provide a threshold above which resistant viruses will be eradicated. We let *R*_2 _be the threshold between Regions 2 and 3.

### The mathematical model

We adapt the mathematical model used from Smith & Wahl [26], to include latently infected cells [27]:

for *t *≠ *t*_*k*_, where

In these equations, *V*_*I *_and *V*_*Y *_denote the wild-type and mutant virus respectively, *V*_*NI *_denotes the non-infectious virus, *T*_*S *_denotes the susceptible CD4^+ ^T cells, *T*_*I *_denotes CD4^+ ^T cells infected by the wild-type virus, denotes *T*_*LI *_denotes CD4^+ ^T cells latently infected by the wild-type virus, *T*_*Y *_denotes CD4T^+ ^T cells infected by the mutant virus, *T*_*LY *_denotes CD4^+ ^T cells latently infected by the mutant virus, *T*_*RY *_denotes the noninfected CD4^+ ^T cells which have absorbed enough drug so the wild-type strain is inhibited, but not enough to prevent infection from the mutant strain, *T*_*RY *_denotes the noninfected CD4^+ ^T cells which have absorbed enough drug to prevent infection from both virus strains, *t *is the time in days, *n*_*I *_is the number of virions produced per infected cell per day, *ω *is the fraction of virions produced per day by an infected CD4^+ ^T cell, *d*_*V *_is the clearance rate of free virus, *r*_*I *_is the rate at which a susceptible cell becomes infected by the wild-type strain, *r*_*Y *_is the rate at which a susceptible cell becomes infected by the mutant strain, *d*_*S *_is the death rate of noninfected CD4^+ ^T cells, *d*_*I *_is the death rate of infected CD4^+^T cells, *ψ *is the proportion of cells which become latently infected, *p*_*L *_is the rate at which latently infected cells become productive, *r*_*P *_is the rate at which the drug inhibits the wild-type T cells when drug concentrations are in Region 2, and *r*_*R *_and *r*_*Q *_are the rates at which the drug inhibits the wild- type and drug-resistant T cells, respectively, when drug concentrations are in Region 3. The constant *λ *is the birth rate of CD4^+ ^T cells, while *m*_*RI *_and *m*_*RY *_are the rates at which the drug is cleared from the intracellular compartment for intermediate and high drug concentrations, respectively. For parameter values and references, see [26].

The dynamics of a drug can be modelled using impulsive differential equations. The exponential decay can be written as a differential equation, where *R*(*t*) is the drug concentration during induction therapy. The dynamics of the drug are

with impulsive conditions, at times *t *= *t*_*k*_,

The rate at which the drug is cleared is *d*_*r *_and *R*^*i *^is the dosage. Assuming a drug is taken at time *t*_*k*_, by the definition of an impulsive effect, we have

### Determining the Region 2 threshold

To find *R*_2_, the Region 2 threshold, we determined the time taken for resistance levels to reach a minimum. The drug levels at this time were evaluated from the antiretroviral effect curves and used as the *R*_2 _threshold. This ensures that, when a drug holiday occurs, resistance levels are guaranteed to be low. Missing several doses increases resistance, but, by using the local minimum values, we ensure that resistance cannot emerge when patients are not taking a drug holiday.

To determine the threshold, note that

since *T*_*Y *_(0) = 0 at the beginning of infection. It follows that the viral load is initially decreasing. If the viral load reaches a minimum at time , then define *R*_2 _= *R*(). This ensures that  < 0 for 0 <*t *<. If the viral load decreases indefinitely, then we could define *R*_2 _to be any value of *R *less than the trough value of the periodic orbit of the drug dynamics. However, this case is not realistic, since the virus does not clear on its own.

We define *R*_1 _to be the value of *R *such

Thus, *R*_1 _= 0.1*R*_2_. See Figure 1.

### Impulsive differential equations

The dynamics of both the wild-type and the resistant strains can be modelled using impulsive differential equations. Impulsive differential equations consist of a system of ordinary differential equations (ODEs), together with difference equations. Between "impulses", *t*_*k*_, the system is continuous, behaving as a system of ODEs. At the impulse points, there is an instantaneous change in state in some or all of the variables. This instantaneous change can occur when certain spatial, temporal or spatio-temporal conditions are met. We refer the interested reader to Bainov & Simeonov [28-30] and Lakshmikantham et al. [31] for more details on the theory of impulsive differential equations.

**Figure 2:**
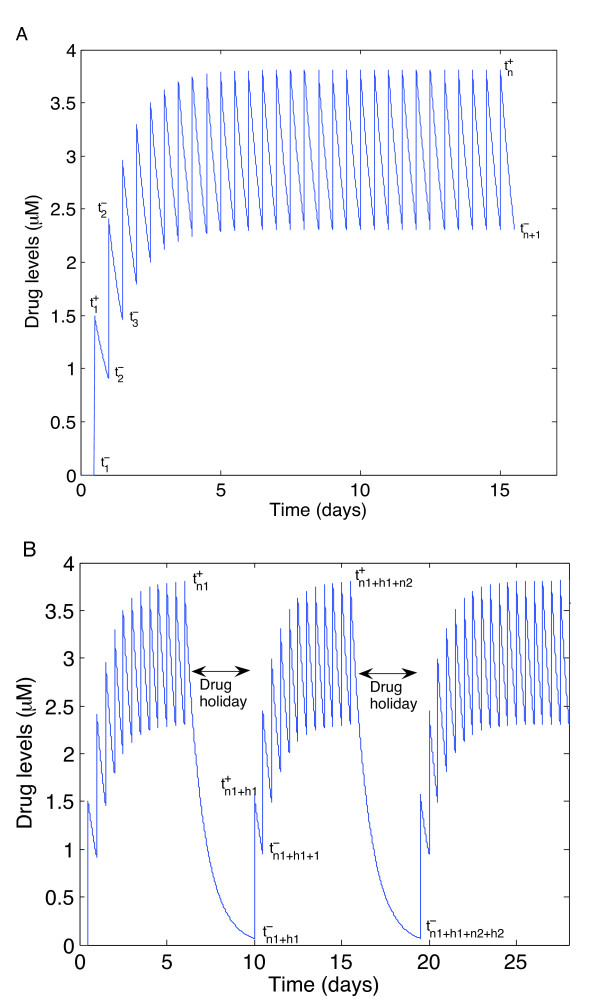
**Drug concentrations**. Drug concentrations using impulsive differential equations. A. Example of drug concentration levels with perfect adherence to therapy. Drug concentration levels fluctuate from lower endpoints  to upper endpoints . Drug concentration levels increase instantaneously after a dose is taken and decrease exponentially between doses. If all doses are taken, drug concentration levels monotonically approach an impulsive orbit. B. Example of fluctuating drug concentration levels when missing drug doses. Once drug concentration levels have reached the impulsive orbit , missing *h *doses results in a long exponential decay. Subsequent adherence returns drug concentration levels to the impulsive periodic orbit before the next drug holiday occurs . In this example, a patient has two drug holidays within a 30 day period.

The change in drug concentration depends on whether a drug is taken or not. There is an instantaneous increase in the drug concentration immediately after a dose is taken and then an exponential decay while the drug is being absorbed in the body. The case of perfect adherence is illustrated in Figure 2A. However, as long as the drug concentration level does not drop below *R*_2_, there is a sufficient amount of drug to control both viral strains. We can thus determine the number of doses that can be missed and the number of doses subsequently taken in order to stay above the *R*_2 _threshold. See Figure 2B.

The differential equations describing the virus and T cells depend on the dynamic behaviour of the drugs. Thus, for example, the rate of change of susceptible T cells decreases in Regions 2 or 3 (at different rates), but not in Region 1. The T cell and virus dynamics are continuous, but their derivatives are not, since those derivatives depend on the drugs, which are discontinuous. Since the drug equations decouple from the remaining equations, we develop theoretical results using the drug equations and apply those results numerically to the entire model.

## Results

### Theoretical results

We used our model to examine the effects of imperfect adherence on the induction phase of IM therapy. First, it is necessary to model perfect adherence to locate the impulsive periodic orbit in the drug levels. This provides a region where the drug concentration level must reach in order to sustain a low viral load. As can be seen in Figure 2, drug levels start at zero during induction therapy (since induction therapy starts at the beginning of drug therapy). Each time a drug is taken, the dose decays at a rate of , where  is the value at which the drug starts to decay instantaneously after the drug is ingested. Since we assume perfect adherence, we get

where *τ *= *t*_*k*+1 _- *t*_*k *_is the (fixed) time between doses for perfect adherence. We thus have

Furthermore,

as *n *→ ∞.

Therefore, assuming perfect adherence, the impulsive orbit has endpoints

Knowing the values of the endpoints for the impulsive orbit after *n *= *n*_1 _doses, we are able to incorporate imperfect adherence and see its effects. In order to avoid Region 2 after missing many doses and to maintain an average drug concentration level within Region 3, we impose conditions to ensure proper therapy. To guarantee successful induction therapy, after the first *n*_1 _doses are taken, we will force the lower endpoint of the drug concentration to be within a tolerance *ε*_1 _of the impulsive orbit. Thus, we require

Once the drug concentration level has reached the impulsive orbit, a patient may take a drug holiday. If *h*_1 _doses are subsequently missed (see Figure 2B), then

In order to avoid Region 2 after *h*_1 _doses are missed, we impose the condition . This will allow us to find the maximum number of doses a patient can miss after being *ε*_1 _away from the impulsive orbit. This results in(1)

After a patient has missed *h*_1 _doses, in order to keep the viral replication low, they must take enough doses, *n*_2_, to return to the impulsive orbit. In the worst-case scenario, the exponential decay has reached Region 2; thus, starting at *R*_2_, we get

After *n*_2 _doses are taken, we must impose a new condition that forces the drug concentration level to be *ε*_2 _away from the impulsive orbit. We need(2)

In order to determine the number of times a patient can miss a fixed amount of doses, we must verify if missing *h*_2 _doses is the same as missing *h*_1 _doses. After missing *h*_2 _doses, we have

Patients are able to miss *h*_2 _doses as long as their drug concentration levels do not drop below Region 2. Thus we repeat the same condition on *h*_2_:

At the end of induction therapy, *k *doses must be taken to ensure that, before the start of maintenance therapy, there is sufficient drug to control viral replication. After missing *h*_2 _doses and assuming we are at Region 2, *k *subsequent doses are taken and the drug level becomes

As can be seen, because we started at the threshold after missing *h*_2 _doses,  as long as *n*_2 _= *k*. Finally, after *k *doses, a patient needs to return to the periodic orbit. Thus, we impose

which is the same as the constraint for *n*_2 _as long as *ε*_2 _= *ε*_3_. If these conditions are satisfied, we are able to guarantee that the drug concentration levels do not enter Region 2 and significant drug resistance will not emerge.

### Imperfect adherence

The number of missable and subsequent doses that must be taken to avoid significant drug resistance for all FDA-approved drugs that are part of a PI-sparing cocktail is shown in Table 1. These are defined by (1) and (2), respectively. However, we stress that these results are theoretical and have not been tested clinically. In particular, it should be noted that pharmacokinetic parameters can vary from patient to patient.

**Table 1:** Missable doses and subsequent adherence.

Drug (units)	*R*^*i *^(*μM*)	*τ *(days)	*T*_1/2 _(hours)	*R*_1 _(*μM*)	*R*_2 _(*μM*)	maximum missable days (theoretical)	minimum subsequent days (theoretical)
Abacavir (ABC)	12	1/2	15	10^-1.0269^	10^-0.0269^	3	7
Didanosine (ddI)	4.65	1/2	25	10^-1.2218^	10^-0.2218^	5	7.5
Emtricitabine (FTC)	7.2	1	39	10^-0.9788^	10^0.0212^	6	17
Lamivudine (3TC)	6	1/2	20	10^-1.1249^	10^-0.1249^	3.5	8.5
Stavudine (d4T)	2.144	1/2	7.5	10^-1.6383^	10^-0.6383^	1	2.5
Tenofivir (TDF)	1.184	1	60	10^-1.5229^	10^-0.5229^	10	24
Zidovudine (ZDV)	4.24	1/3	7	10^-1.6021^	10^-0.6021^	1.33	2.67
Delavirdine (DLV)	26.6	1/3	5.8	10^-1.4559^	10^-0.4559^	1.67	2.67
Efavirenz (EFV)	12.9	1	45	10^-0.8356^	10^0.1644^	9	22
Nevirapine (NVP)	7.5	1/2	27	10^-1.0088^	10^-0.0088^	5	12.5

Summary of data and theoretical results for reverse transcriptase inhibitors used for FDA-approved triple-drug therapy. All results are calculated with a mutant that exhibits 10-fold resistance to the drug. The decay rate *d*_*r *_was calculated using the formula *d*_*r *_= 24 log(2)/*T*_1/2_, where *T*_1/2 _is the half-life. The last two columns are the number of doses that may be missed before significant drug resistance emerges and the number that must subsequently be taken to return to within 0.01 *μM *of perfect adherence. We used the intracellular half-life for each drug, if known. Data for Column 1 was taken from [41-48], and data from Columns 2 and 3 were taken from the Department of Health and Human Services 2008 Guidelines for the Use of Antiretroviral Agents in HIV-1-Infected Adults and Adolescents [37]

There are fifteen FDA-approved PI-sparing triple-drug cocktails, for which we calculated (a) the initial number of doses that must be taken to be within a prescribed tolerance of perfect adherence, (b) the number of doses that could be missed without significant drug resistance emerging and (c) the number of doses that must be taken subsequently.

**Figure 3:**
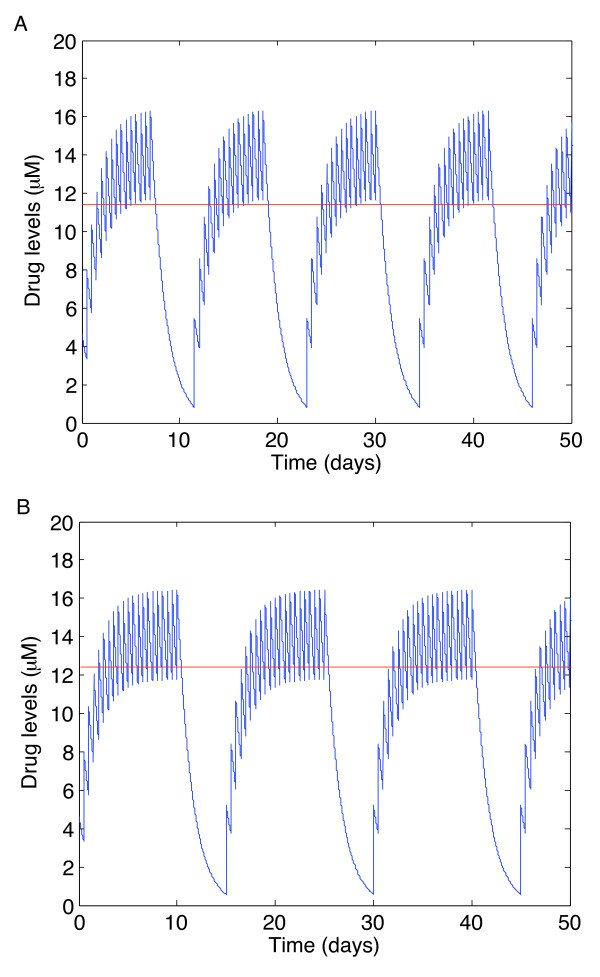
**Determining the prescribed tolerance**. Difference between a prescribed tolerance of (A) 0.1 *μM *and (B) 0.01 *μM *for the reverse transcriptase inhibitor Didanosine (ddI). The red line plotted on both graphs is the average drug concentration while taking drug holidays. This was calculated using the data from Table 2. The average drug concentration in (A) is around 11 *μ*M and has not reached the trough values when drug holidays are excluded, whereas the average drug concentration in (B) is around 12 *μ*M and thus exceeds the trough values during therapy.

To determine the value of the prescribed tolerance, we examined two possibilities: a tolerance of 0.1 *μM *and a tolerance of 0.01 *μM*. That is, the number of doses is considered sufficient if the trough value of the periodic orbit of the drug dynamics is within 0.01 *μM *of the trough value of therapy without drug holidays. We imposed a further condition: that the mean drug concentration be larger than the trough value of drugs when no drug holidays are taken. This is illustrated in Figure 3. This ensures that, over the length of the entire induction phase, drugs are maintained at sufficiently high levels (see [26] for more discussion). In Figure 3A, using a tolerance of 0.1 *μM*, the overall mean drug concentration is below the trough value during therapy. Using a tolerance of 0.01 *μM*, as shown in Figure 3B, shows that the overall mean drug concentration is above the trough value during therapy.

**Table 2:** Number of drug holidays.

FDA-approved combination			Number of drug holidays (theoretical)	Length of each holiday (days)	Minimum subsequent therapy (days)
ABC*	3TC	NVP	16	3	7
ABC*	3TC	EFV	16	3	7
TDF	3TC*	EFV	14	3.5	8.5
ddI	3TC*	EFV	14	3.5	8.5
d4T*	3TC	EFV	50	1	2.5
d4T*	3TC	NVP	50	1	2.5
ddI*	FTC	EFV	13	5	7.5
TDF	FTC*	EFV	7	6	17
TDF	FTC	NVP*	9	5	12.5
ZDV*	3TC	ABC	44	1.33	2.66
ZDV*	3TC	EFV	44	1.33	2.66
ZDV*	3TC	NVP	44	1.33	2.66
ZDV*	3TC	TDF	44	1.33	2.66
ZDV*	DLV	3TC	44	1.33	2.66
ZDV*	DLV	ddI	44	1.33	2.66

Summary of all FDA-approved, PI-sparing triple-drug combinations. Drugs marked with an asterisk are the drug in their respective cocktail with the least number of doses that may theoretically be missed (see Table 1). The number of drug holidays, the length of each holiday and the minimum number of subsequent days of strict adherence is thus calculated from this drug's missable and subsequent doses.

For the fifteen FDA-approved PI-sparing triple-drug cocktails, we identified the "weakest" drugs in each cocktail; ie, those for which the least number of doses can be missed. These drugs are Abacavir (ABC), Lamivudine (3TC), Stavudine (d4T), Emtricitabine (FTC), Zidovudine (ZDF), Didanosine (ddI) and Nevirapine (NVP). Thus, for each cocktail, the maximal number of missable doses is the same as that of its "weakest" drug. By combining the steps in (b) and (c) above, it was possible to theoretically calculate the number of drug holidays that could be taken during the inductive phase, based on the regimen for the "weakest" drug. See Table 2.

Since the minimum number of doses required to be taken and the maximum number of doses allowed to be missed follow a reliable pattern, we can extend this to fit into a baseline induction phase of 180 days [20]. This means, for example, that a patient taking the triple-drug cocktail FTC/TDF/EFV can theoretically have a 6 day holiday, as long as each holiday is followed by 17 days of perfect adherence; patients can take seven such holidays during the induction phase, and are thus able to miss a total of 42 days out of 180. A patient taking ABC/3TC/NVP can theoretically have sixteen drug holidays of 3 days each in a 180 day period, as long as each holiday is immediately followed by a 7 day period of strict adherence.

### Numerical simulations

In order to determine the long-term effects of taking the prescribed drug holidays, we simulated the worst-case scenario: monotherapy to the "weakest" drug in each combination from Table 2. This has the effect of overestimating the development of resistance: if no resistance is predicted to emerge during monotherapy, then it is unlikely to emerge during combination therapy. Conversely, if resistance does emerge during monotherapy, then there is no guarantee that it would emerge during combination therapy, due to the presence of the other two drugs.

We considered an extinction threshold of 2 × 10^-4 ^virions/mL. This corresponds to the concentration at which the virus falls below 1 per body. Thus, missing the maximum number of doses would theoretically lead to extinction of both strains (at least up to the level of detection), whereas missing more doses does not. However, it should be noted that we did not curtail the viral dynamics at this threshold.

We used the model in Section (describing the dynamic interaction between virus, T cells and drugs) and the calculations in Section (summarised in Tables 1 and 2) to illustrate our theoretical results. In order to demonstrate the effects of taking the prescribed drug holidays, we first ran simulations where patients missed the maximum number of doses and then took the required number of subsequent doses; this cycle was repeated for 180 days. Next, we ran the same simulations, with the same parameters, except that one additional dose of the drug was skipped during each drug holiday.

**Figure 4:**
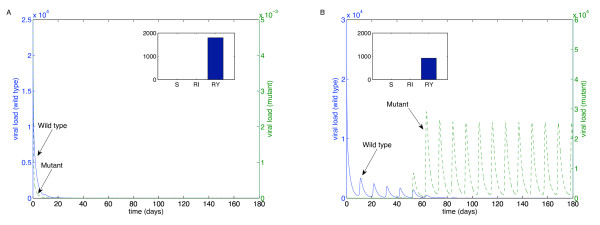
**Adherence to ABC monotherapy**. A. Long-term effects of adherence to ABC monotherapy, using the prescribed adherence breaks. The wild type (solid blue curve, left axes) and mutant (dashed green curve, right axes) populations are shown. The overall effect of the mutant remains low. Parameters used, in addition to those in Table 1, were *n*_*I *_= 262.5 day^-1^, *ω *= 0.7, *r*_*I *_= 0.01 day^-1^, *r*_*Y *_= 0.001 day^-1^, *d*_*V *_= 3 day^-1^, *d*_*S *_= 0.1 day^-1^, *d*_*I *_= 0.5 day^-1^, *ψ *= 0.2, *p*_*L *_= 0.05, *r*_*R *_= *r*_*Q *_= 80 *μM*^-1^, day^-1^, *λ *= 180 cells *μL*^-1 ^and *m*_*RI *_= *m*_*RY *_= log(2) day^-1^. Initial conditions were *V*_*I *_(0) = 22000 virions mL^-1^, *V*_*Y *_(0) = 5 × 10^-3 ^virions mL^-1^, *T*_*S *_(0) = 1000 cells day^-1 ^and all other initial conditions were zero. B. The effects of missing one extra dose per drug holiday. The proportions of each type of uninfected T cell at the end of the simulation are shown in the insets.

**Figure 5:**
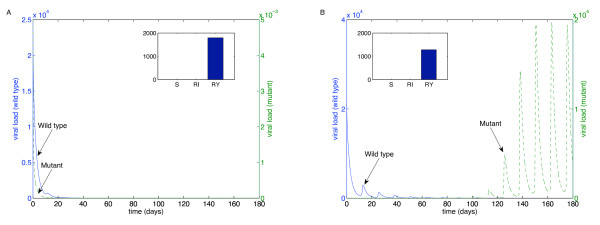
**Adherence to 3TC monotherapy**. A. Long-term effects of adherence to 3TC monotherapy, using prescribed adherence breaks. B. The effects of missing one extra dose. Drug parameters are as in Table 1, while all other parameters are as in Figure 4. The proportions of each type of uninfected T cell at the end of the simulation are shown in the insets.

**Figure 6:**
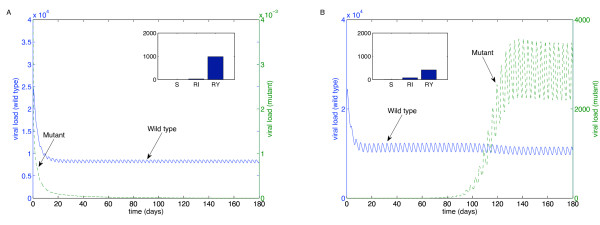
**Adherence to d4T monotherapy**. A. Long-term effects of adherence to d4T monotherapy, using prescribed adherence breaks. B. The effects of missing one extra dose. Drug parameters are as in Table 1, while all other parameters are as in Figure 4. The proportions of each type of uninfected T cell at the end of the simulation are shown in the insets.

**Figure 7:**
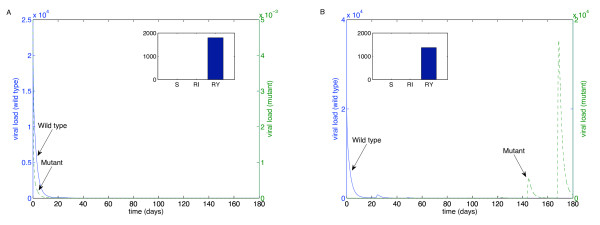
**Adherence to FTC monotherapy**. A. Long-term effects of adherence to FTC monotherapy, using prescribed adherence breaks. B. The effects of missing one extra dose. Drug parameters are as in Table 1, while all other parameters are as in Figure 4. The proportions of each type of uninfected T cell at the end of the simulation are shown in the insets.

**Figure 8:**
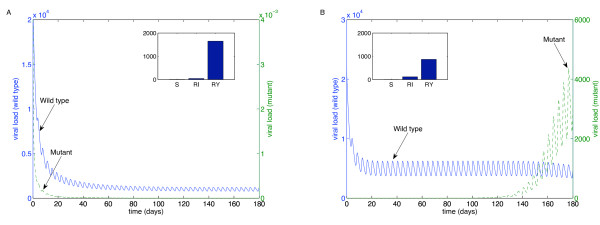
**Adherence to ZDV monotherapy**. A. Long-term effects of adherence to ZDV monotherapy, using prescribed adherence breaks. B. The effects of missing one extra dose. Drug parameters are as in Table 1, while all other parameters are as in Figure 4. The proportions of each type of uninfected T cell at the end of the simulation are shown in the insets.

**Figure 9:**
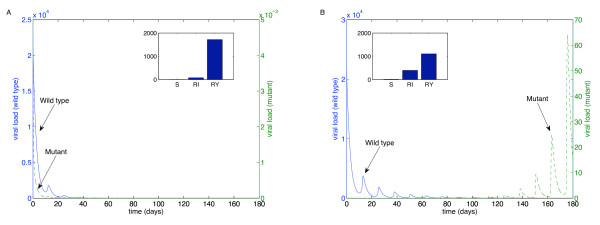
**Adherence to ddI monotherapy**. A. Long-term effects of adherence to ddI monotherapy, using prescribed adherence breaks. B. The effects of missing one extra dose. Drug parameters are as in Table 1, while all other parameters are as in Figure 4. The proportions of each type of uninfected T cell at the end of the simulation are shown in the insets.

**Figure 10:**
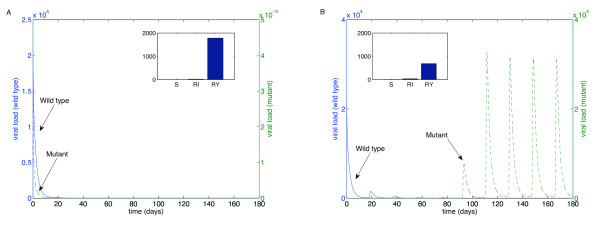
**Adherence to NVP monotherapy**. A. Long-term effects of adherence to NVP monotherapy, using prescribed adherence breaks. B. The effects of missing one extra dose. Drug parameters as as in Table 1, while all other parameters are as in Figure 4. In this case, both strains are controlled. Inset: The effects of missing three extra doses. In this case, the wild-type strain is controlled, but the resistant strain emerges. The proportions of each type of uninfected T cell at the end of the simulation are shown in the insets.

We performed these simulations for each of the "weakest" drugs identified in Table 2: ABC (Figure 4), 3TC (Figure 5), d4T (Figure 6), FTC (Figure 7), ZDV (Figure 8), ddI (Figure 9) and NVP (Figure 10). The first figure in each case illustrates the case of missing the maximal drug holiday and taking the minimum number of subsequent doses. The second figure in each case illustrates the same case, except that one additional dose was missed during each drug holiday. The exception is NVP, in which resistance did not emerge until three extra doses were missed (Figure 10B, inset).

For the first case, the wild-type virus oscillated at low levels during each drug holiday, but significant levels of resistance did not appear. Thus, taking the required number of doses successfully keeps the mutant strain at low levels. Conversely, missing one extra dose per holiday (three in the case of NVP) resulted in a significant buildup of resistance by the end of the induction phase.

In this case, there is a tremendous increase in the mutant strain by the end of the inductive phase, indicating that therapy has failed. Resistance to Abacavir increases from 10^-3 ^to 10^4^; resistance to Lamivudine increased from less than 10^-3 ^to 10^4^; resistance to Stavudine increased from 10^-3 ^to 10^3^; resistance to Emtricitabine increased from 10^-3 ^to 10^4^; resistance to Zidovudine increased from 10^-3 ^to 10^3^; resistance to Didanosine increased from 10^-3 ^to 10^2^; and resistance to Nevirapine increased from 10^-3 ^to 10^4^.

### Comparison with clinical results

A number of studies have attempted to characterise the safety of regular (and irregular) treatment interruptions, generally referred to as structured treatment interruptions (STIs). Pai *et al*. [22] summarised the to-date evidence of STIs in patients with chronic unsuppressed HIV infection due to drug-resistant HIV. They concluded that there were no significant virologic or immunologic benefit to STIs and that there is evidence that STIs have a prolonged negative impact on CD4 response and other disease events.

Subsequently, the SMART trial [32] examined CD4^+ ^guided interruptions, of an average duration of 16 months. The DART trial [33] examined fixed 12 week interruptions. Both trials showed no benefit to these treatment interruptions. Indeed, the SMART trial was halted prematurely, due to significant morbidity and mortality among participants. Holkmann *et al*. [34] reported a two-fold risk of AIDS or death for patients who underwent treatment interruptions that lasted three months or longer.

It should be noted that all these trials involved lengthy periods of treatment interruption, of the order of weeks. Our results here recommend signficantly shorter periods of treatment interruption, of the order of days. Furthermore, our results predict significant increase in resistance if these periods are exceeded, consistent with the results from the majority of trials.

Shorter treatment interruptions have also been investigated. A study comparing interruptions of less than 7 days compared to longer interruptions showed that only 5% of men who discontinued HAART for short periods increased their HIV RNA. Conversely, men with longer interruptions had significantly higher rates (35.7 of HIV RNA increase [35]. Another study investigating cycles of 2-6 week fixed interruptions observed no clinically significant benefit with regard to viral suppression when off HAART, but also observed no evidence for an increase of viral resistance among patients undergoing repeated interruptions [36].

Recently, a pilot study examining five days on, two days off (FOTO) followed patients for 48 weeks [25]. Virologic suppression was maintained in 89.6% of patients. Combinations included 3TC/TDF/EFV, ABC/TDF/EFV, ddI/3TC/EFV and ABC/ddI/TDF/EFV; 100% of subjects on EFV- based regimens on the FOTO treatment schedule maintained virologic suppression at weeks 24 and 48. Combinations also included nevirapine- based regimens where one subject, on NVP/ABC/3TC/ZDV, had viral rebound at week 12 that was confirmed at week 16 on the FOTO schedule. It was also noted that 30% of the subjects on nevirapine-based regimens had blips of viral increase during therapy. Other combinations included TDF/3TC/NVP, ZDV/3TC/NVP, d4T/3TC/TDF/NVP. They also observed excellent adherence to the FOTO treatment schedule and a strong preference for this schedule compared to HAART. None of the observed rebounds in viral load were associated with the reported adherence of more than 2 days off therapy.

These preliminary results are in line with our theoretical recommendations. For regimens that include EFV-based regimens, all therapies included NRTIs and NNRTIs that we recommend a maximum of more than 2 days per drug holiday, followed by at least 5 days of subsequent therapy (Table 2). The NVP-based regimen with viral rebound included ZDV; our results predict that drug holidays on such a regimen should be no longer than 1.33 days (Figure 8). The three other NVP-based regimens with viral blips included ZDV and d4T; our results predict that neither would allow drug holidays as long as two days (Table 1).

### Sensitivity to variations

Since individual patients may respond differently to drugs, we explore the sensitivity of the number of missable doses to variations in parameters. The number of missable doses depends on the dosing interval, the drug decay rate, the drug concentration, the Region 2 threshold and the number of initial doses, which itself depends on the prescribed tolerance. Since we have already explored variations in the dosing interval and the prescribed decay rate, we now examine the variation with respect to the other parameters.

**Figure 11:**
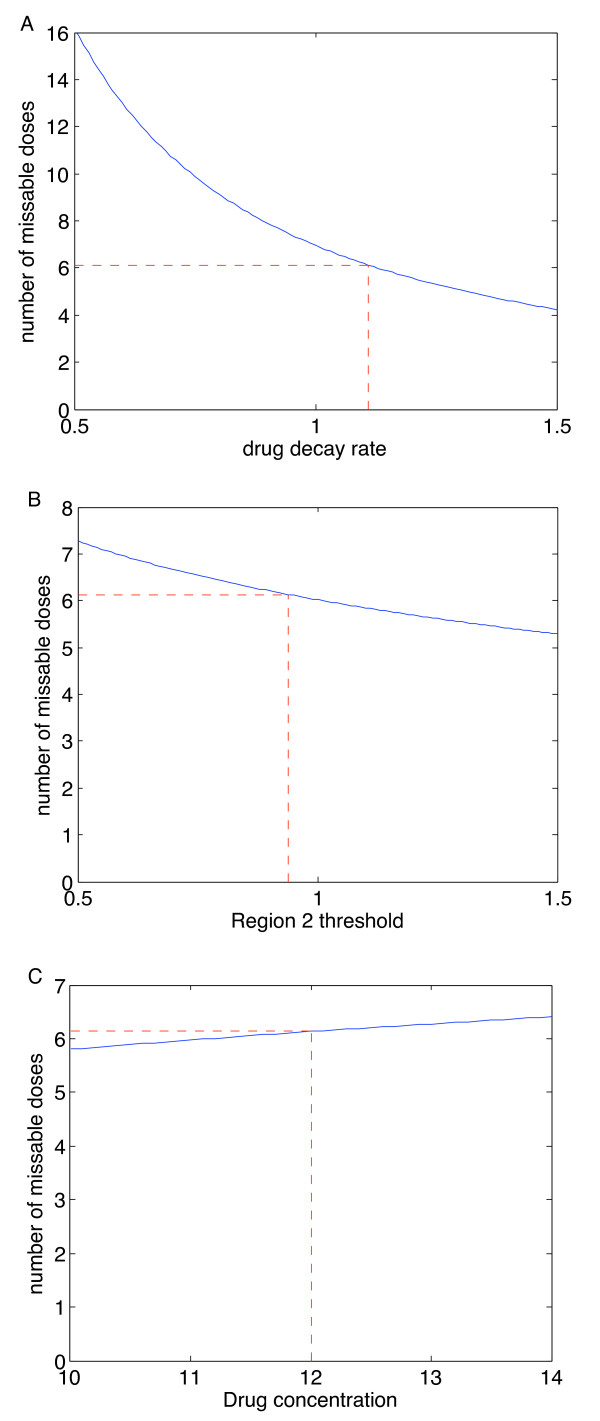
**Sensitivity to other parameters**. Sensitivity of length of drug holiday to (A) the drug decay rate, *d*_*r*_, (B) the Region 2 threshold, *R*_2_, and (C) the drug concentration, *R*^*i*^. Dashed lines indicate values used in our calculations. This example is for ABC.

Figure 11 demonstrates the effect of variations in the drug decay rate, the Region 2 threshold and the drug concentration. Since the slope of the curves is low for the second and third figures, we conclude that the results are not highly sensitive to variations in the Region 2 threshold or the drug concentration, although small fluctuations may decrease the number of missable days (Figure 11B and Figure 11C). The outcome is more sensitive to variations in the drug decay rates, but is still not highly sensitive (Figure 11A).

## Discussion

It is vital to provide HIV patients with an effective drug regimen. Not only is it important that the drugs have a high effcacy, but it is also important that patients follow a regimen that will benefit both their mental and physical states. Since there are such a large number of patients who are unable to take their drugs regularly, it is important to understand the impact of drug holidays upon a patient's ability to control the virus. Induction therapy provides patients with the chance to submit to a very strict, but short, period of intense drug taking, followed by a long period of less-restrictive and more-relaxed therapy (maintenance therapy). We have demonstrated the effects of taking drug holidays during induction therapy. Instead of taking drugs two to three times a day for the entire length of the induction period, we were able to show that a patient can have drug holidays with sometimes as much as six days off each time. This form of treatment allows patients to take drug holidays with very little negative effect.

However, missing more doses than stated can highly affect the amount of resistant virus created. We have demonstrated that there is a large increase of mutant virus by simply missing one extra dose during each drug holiday (three for the TDF- FTC-NVP combination). Induction therapy with partial adherence works as long as a patient does not exceed the maximum length of the drug holiday; if they follow the prescribed regime, they can control the effects of drug resistance. It should be noted that Curlin *et al*. [20] showed that an induction phase on the order of 180-days was ideal for a triple-drug therapy including two RTI-like drugs and one PI-like drug. Since the results for a triple-drug therapy including three RTIs do not show a dramatic increase in resistant virus while taking the patterns suggested, we used an 180 day induction phase as a baseline.

These results apply to the fifteen FDA-approved, PI-sparing triple-drug cocktails, but simulations were only performed for the drugs with the least number of missable doses: Abacavir, Lamivudine, Stavudine, Emtricitabine, Zidovudine, Didanosine and Nevirapine. Missing one extra doses at the end of each drug holiday (three for Nevirapine) drastically increases the amount of resistant virus. However, it should be noted that the simulations were for monotherapy only and thus, in a triple-drug cocktail, the remaining two drugs inure against resistance.

Efavirenz and nevirapine only require a single mutation to confer resistance, and cross resistance affecting these three NNRTIs is common [37]. Both Lamivudine and Emtricitabine select for the M184V resistance mutation, which confers high-level resistance to both drugs, a modest decrease in susceptibility to Didanosine and Abacavir, and improved susceptibility to Zidovudine, Stavudine and Tenofovir [38]. It should be noted that our model assumes that the mutant is always present. By simulating the results for monotherapy, we illustrated the worst-case scenario; this is illustrated by Figure 10B, which shows that missing one extra dose per holiday is not disastrous; in this example, the mutant only takes hold when three extra doses are missed. Thus, our results are more conservative than is strictly necessary.

Double mutation happens less frequently; emergence of the M184V mutation is less frequent with Tenofovir/Emtricitabine than with Zidovudine/Lamivudine, while selection of the Lamivudine-associated M184V mutation to the Zidovudine/Lamivudine combination has been associated with increased susceptibility to Zidovudine [37]. It follows that, when the combinations are taken synchronously, the selection of mutants will be signficantly less likely than under monotherapy.

Discontinuous dosing is, of course, not realistic. There is a delay, the time-to- peak, between taking a drug and it reaching peak values in cells. Consequently, estimates based on maximal concentrations and terminal plasma half-lives could overestimate drug exposure. However, such delays can be approximated by an instantaneous change if the time-to-peak is sufficiently short, compared to the time between doses. This approximation has been shown to be robust, even for quite large delays [39].

Other limitations to our model are the assumption that the CD4^+ ^pool of lymphocytes is the most significant source of HIV infection and that maintaining drug concentrations at clinical levels results in maximal control of virus replication. However, not all HIV-susceptible tissues are equally susceptible to antiretroviral drugs. For example, lymphoid cells in the gut are not completely suppressed [40]. These and other reservoirs will contribute to the long-term generation of virus particles, both during therapy and while undergoing a drug holiday. The relative rates of mutation or selection of resistant viruses for the various drugs are modelled via the choice of infection rate, *r*_*Y*_, compared to the infection rate, *r*_*I*_, for the wild-type strain. For numerical simulations, we used the intracellular half-life of each drug, if known; in the case of nucleosides, it is the cellular concentration of active nucleotide that is responsible for inhibition of viral reverse transcription. Furthermore, we assume that all tissues harbouring HIV are exposed to the same concentration of drug.

Previously [13], we showed how many doses can be missed for each PI- sparing drug, for only a single drug holiday during any given therapy. Here, we extend this to the case of more than one drug holiday.

Furthermore, all previous mathematical models of adherence considered therapy without an endpoint. Since induction therapy only occurs for a finite time, we have to consider the viral load when induction therapy ends. In particular, if a drug holiday coincided with the end of induction therapy, then the induction phase would functionally have ended at an earlier time and may thus be significantly less effective. Some of the key differences between our earlier work and the results provided here occur due to the fact that here we use 10-fold resistance, rather than 50- fold resistance; multiple holidays occur during a finite time interval; and the tolerance used was 0.01 *μM *instead of 1% of the minimum value of periodic orbit; the tolerance we used here is more conservative.

Future work will investigate the effects of imperfect adherence to triple-drug cocktails involving protease inhibitors. We will also investigate the compounding effects of combination therapy in slowing the emergence of resistance and the effect of inter-individual variances in pharmacokinetics. Our modelling process could also be extended to additional treatment scenarios in which patients might be tempted to take drug holidays due to a high pill burden, such as booster therapies or the initial year of HAART.

## Conclusions

Using readily available pharmacokinetic data, we can theoretically determine the maximal length of drug holidays and the number of subsequent doses that must be taken. Since the induction phase lasts for a finite time, we can thus determine how many drug holidays can be taken within a 180-day induction period. Our theoretical results are in line with recent results concerning five-days-on/two-days-off (FOTO) for most cocktails, suggesting that drug holidays may be limited to very short breaks, rather than the longer holidays previously examined.

We thus conclude that induction therapy with partial adherence is tolerable, but the outcome depends on the drug cocktail. We have also demonstrated a robust method by which to determine therapy guidelines for patients who are unable or unwilling to adhere completely. Treatment interruptions, if they occur, must be short and followed by a strict period of dose taking. Thus, while continuous therapy is preferable, FOTO therapy is acceptable for all RTI cocktails except those containing ZDV, d4T or DLV, which can only tolerate extremely short drug holidays.

## Competing interests

The authors declare that they have no competing interests.

## Authors' contributions

Both authors wrote the manuscript and performed numerical simulations. RJS designed the study, while REM performed the mathematical analysis. Both authors read and approved the final manuscript.

## Pre-publication history

The pre-publication history for this paper can be accessed here:

http://www.biomedcentral.com/1471-2334/10/6/prepub
